# Effects of combined Baduanjin and cognitive training on cognitive function in cognitively normal older adults

**DOI:** 10.3389/fpsyg.2026.1903040

**Published:** 2026-08-14

**Authors:** Cui Li, Yujiao He, Xinzhe Zhang, Xuerui Chen, Xueyan Zhang, Heng Zhang, Haoxuan Zhang, Boyang Fu, Yapeng Wei, Jianrui Li, Nan Wang

**Affiliations:** School of Kinesiology and Physical Education, Zhengzhou University, Zhengzhou, Henan, China

**Keywords:** aging, cognitive decline, cognitive intervention, mind–body exercise, prevention

## Abstract

**Objectives:**

Preventing cognitive decline is a major challenge in aging research. While mind–body exercises and cognitive training each enhance cognitive function, their combined impact on cognitively normal older adults remains unclear. This study examined whether Baduanjin exercise and cognitive training exert interactive effects on global and domain-specific cognitive function.

**Methods:**

In a 12-week randomized controlled trial with a 2 × 2 factorial design, 162 community-dwelling adults aged 60–75 years with normal cognitive function were assigned to four groups: control (CON), Baduanjin (BG), cognitive training (CT), and combined intervention (BG + CT). Global cognition, memory, attention, executive function, language, and visuospatial ability were assessed at baseline and post-intervention. Changes within-group and main and interaction effects were analyzed.

**Results:**

One hundred thirty-eight participants completed the intervention. All intervention groups showed significant cognitive improvements from baseline. BG + CT demonstrated the most consistent improvements, including unique significant gains in long-delay memory. Factorial analyses revealed significant main effects of both Baduanjin and cognitive training on global cognition and multiple cognitive domains (*p* < 0.05), with notable interaction effects between the two interventions. Simple effects analyses confirmed that BG + CT led to greater improvements than individual interventions, particularly in long-delay memory, executive function, language, and visuospatial ability.

**Conclusion:**

Combined Baduanjin and cognitive training was associated with greater cognitive benefits than single interventions in cognitively normal older adults. These findings support the potential value of integrated behavioral interventions for maintaining cognitive health in later life.

## 1 Introduction

Population aging is a significant global phenomenon (Jakovljevic et al., 2018) and is accompanied by a progressive, widespread decline in age-related cognitive abilities (Dar et al., 2025). Even in older adults deemed cognitively normal, gradual declines in cognitive functions such as memory, attention, and executive function are commonly observed as part of the normal aging process (Kauppi et al., 2020). These subtle changes may negatively affect daily functioning, psychological well-being, and quality of life, and may increase the long-term risk of developing mild cognitive impairment (MCI) or dementia (Herrmann et al., 2025). Therefore, identifying safe and effective non-pharmacological interventions to mitigate cognitive decline in cognitively normal older individuals has emerged as a core priority in public health (Liu-Ambrose et al., 2019).

Cognitive aging is characterized as a continuous process, not as a discrete clinical condition. The stage of normal cognition represents a critical window for preventive intervention, during which cognitive plasticity and cognitive reserve remain relatively preserved (Cheng, 2016; Sun and Alkon, 2024). The intervention measures implemented during this period may help to slow down age-related cognitive decline, enhance cognitive reserve, and potentially reduce the risk of subsequent cognitive impairments (Lisko et al., 2021; Reijnders et al., 2013). Compared with interventions targeting individuals with established cognitive deficits, preventive approaches for cognitively normal older adults may offer broader and more sustainable benefits (Livingston et al., 2020; van der Flier et al., 2023).

Mind–body exercises have received growing attention for their potential cognitive benefits in older adults (Ye et al., 2021; Zhang et al., 2023). Baduanjin, a traditional Chinese Qigong exercise characterized by slow, regulated breathing and focused attention (Wang et al., 2021), has been shown to improve global cognitive function and specific cognitive domains, such as memory (Hong et al., 2024; Yu et al., 2021) and executive function (Lin S. et al., 2022; Wang et al., 2024). These improvements may be mediated through multiple mechanisms, including increased cerebral blood flow, reduced psychological stress, and enhanced mind–body integration (Hong et al., 2024; Lin et al., 2023; Shen et al., 2024). In parallel, cognitive training has demonstrated efficacy in improving targeted cognitive domains, such as attention, memory, and executive function, by repeatedly engaging cognitive processes and promoting neuroplasticity (Fang et al., 2024; Pieramico et al., 2012). Recent studies suggest that combining physical exercise with cognitive training may produce greater cognitive benefits than either intervention alone for adults with mild cognitive impairment and dementia (Castellote-Caballero et al., 2024; Cheng, 2016; Xue et al., 2023). Physical exercise may provide a favorable neurobiological environment by enhancing cerebral perfusion and neurotrophic factor expression (Hong et al., 2024; Lin et al., 2023; Zhang et al., 2024), whereas cognitive training directly stimulates cognitive processing and neural networks (Turnbull et al., 2022). From this perspective, integrating Baduanjin with cognitive training may exert additive and complementary effects by simultaneously targeting physical, emotional, and cognitive aspects of aging (Chen et al., 2023). However, most existing studies have focused on single-modality interventions, predominantly targeting individuals with mild cognitive impairment or dementia (Orgeta et al., 2020; Sung et al., 2023; Wang et al., 2021; Wang et al., 2024; Zhang et al., 2023). Empirical evidence regarding the effects of combined interventions on cognitively normal older adults remains scarce.

Therefore, this study aims to explore the effects of Baduanjin exercise, cognitive training, and their combined intervention on overall cognitive function and specific cognitive domains (including memory, attention, executive function, language, and visuospatial ability) in cognitively normal older individuals. Using a four-group randomized controlled design, cognitive performance was assessed before and after a three-month intervention period. We hypothesized that both Baduanjin and cognitive training would improve cognitive function compared with the control condition, and that the combined intervention would yield greater improvements in global and domain-specific cognitive functions than either single intervention alone.

## 2 Methods

### 2.1 Experimental design

**Figure 1:**
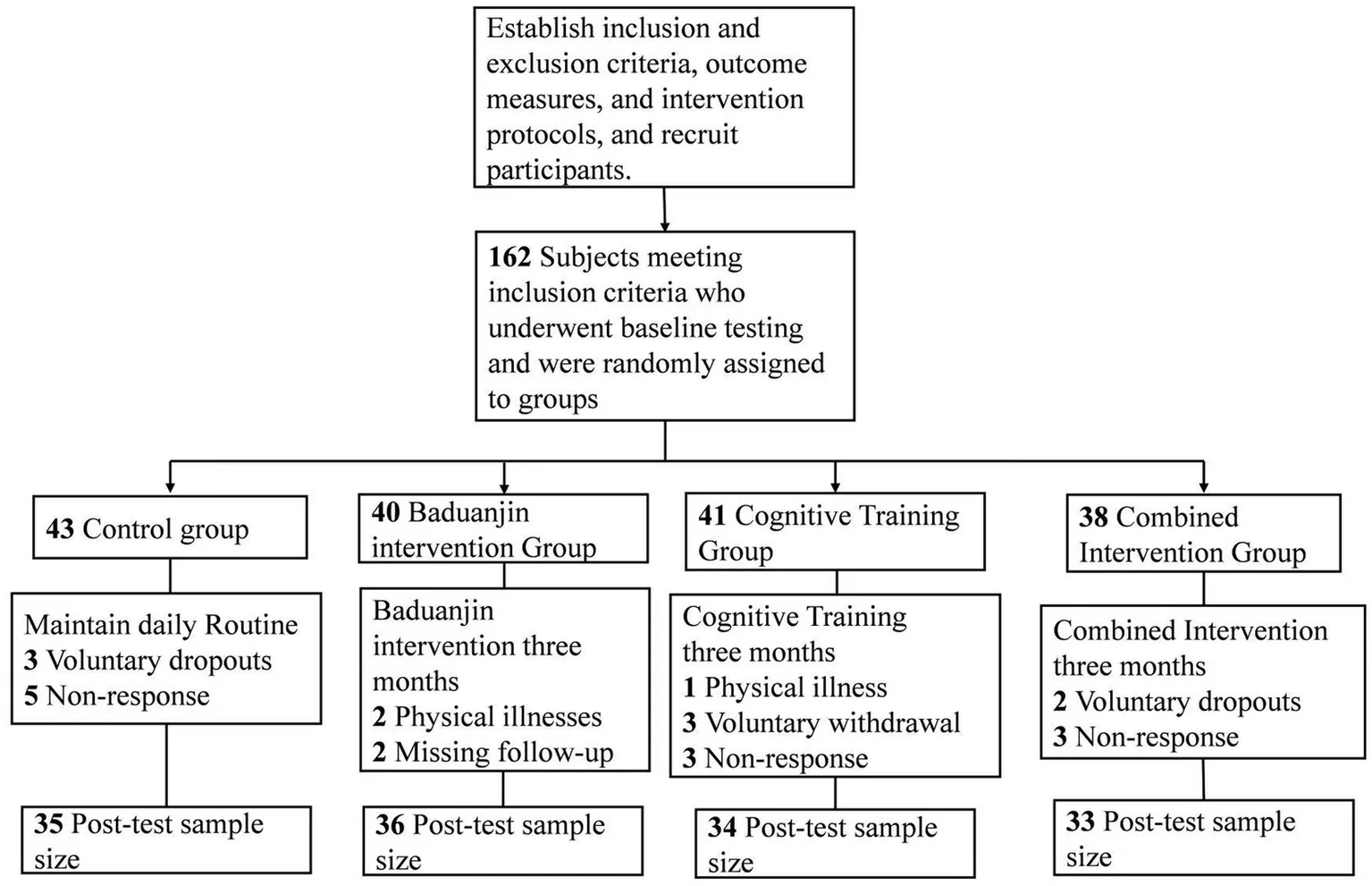
Flow diagram of participant enrollment, intervention allocation, and follow-up. A total of 162 participants meeting the inclusion criteria completed baseline assessments and were randomly assigned to the control group, Baduanjin exercise group, cognitive training group, or combined intervention group. Reasons for attrition during the intervention period included voluntary withdrawal, physical illness, and non-response. A total of 138 participants completed the intervention.

This study employed a 2 × 2 randomized controlled trial to explore the effects of Baduanjin exercise, cognitive training, and their combined intervention on the cognitive functions of older adults living in the community. According to inclusion/exclusion criteria, 162 participants were recruited. They were randomly assigned via computer-generated random numbers to four groups: control (*n* = 43), Baduanjin (*n* = 40), cognitive training (*n* = 41), and combined intervention (*n* = 38). Outcome assessments were conducted at baseline and immediately after the three-month intervention period by trained assessors who were blinded to group allocation. Intervention delivery and outcome assessments were conducted by different research staff to minimize potential assessment bias. Ultimately, 138 participants completed the study, with dropouts attributed to voluntary withdrawal, loss to follow-up, or health-related reasons. The study flowchart is shown in Figure 1.

### 2.2 Participants

Participants were recruited from six communities in the High-Tech Development Zone of Zhengzhou, Henan Province, China, through community health lectures and recruitment posters displayed at community activity centers and bulletin boards. Interested individuals were screened by trained research staff against the inclusion and exclusion criteria, and eligible participants provided written informed consent before the baseline assessment. Sample size estimation was performed using G^*^Power 3.1 software based on analysis of variance (four groups), assuming a medium effect size (ƒ = 0.25), a significance level of *α* = 0.05, and a statistical power of 0.80 (Kang, 2021). The estimated minimum sample size was 128 participants. Considering potential attrition, 162 participants were recruited and randomly allocated to the four study groups. All participants provided written informed consent before participating. This study was reviewed and approved by the Ethical Committee of the School of Physical Education, Zhengzhou University (Number: ZZUIRB2024-04).

The study’s inclusion criteria were as follows: (1) participants aged between 60 and 75 years; (2) independence in activities of daily living; (3) the ability to ambulate independently; and (4) a score of ≥ 26 on the Montreal Cognitive Assessment (MoCA), with an additional point added for individuals with ≤ 12 years of education. Exclusion criteria encompassed: a diagnosis of cognitive dysfunction or neurodegenerative disease; severe psychiatric disorders; the use of medications known to affect cognitive function; severe visual or auditory impairment; communication difficulties; mobility limitations; and any contraindication to physical exercise.

### 2.3 Group-specific interventions

Participants in the Baduanjin group engaged in supervised Baduanjin training for 12 weeks, with three 60-min sessions per week. Each session consisted of a 5-min warm-up, 50 min of Baduanjin practice, and a 5-min cool-down. The intervention employed standardized Baduanjin routines (Wang et al., 2024), emphasizing coordinated movements, controlled breathing, and focused attention. The Baduanjin intervention was delivered under the supervision of two certified instructors holding the Social Sports Instructor certification in Qigong. One instructor led the exercise sessions, while the second instructor provided on-site supervision and corrective feedback to ensure accurate and consistent movements.

Participants in the cognitive training group received structured cognitive training three times per week for 12 weeks, with each session lasting 60 min. The training was conducted in a group format by trained research staff following standardized protocols, in accordance with guidelines established by cognitive training experts. Task difficulty was progressively adjusted based on participant performance. The training content targeted five cognitive domains: (1) memory training, including word chain games, riddles, song recognition, paired associative learning, face-name memorization, and story recall; (2) attention training, including Schulte grid exercises, visual tracking, “spot-the-difference” games, forward and backward digit repetition, and digit-symbol substitution games; (3) language training, including tongue twisters, word chain games, general knowledge quizzes, and poetry recitation; (4) executive function training, including reasoning puzzles, jigsaw puzzles, Sudoku, “do the opposite” commands, and manual crafting activities; (5) visuospatial training, including maze navigation, direction identification, visual tracking tasks, route description, and block design/spatial combination tasks.

Participants in the combined intervention group received both Baduanjin exercise and cognitive training over 12 weeks, with the two interventions administered on alternate days, three times per week each, for a total of six 60-min sessions per week.

Participants in the control group continued with their regular daily activities throughout the study period and did not partake in any structured physical exercise or cognitive training interventions.

### 2.4 Outcomes

Assessments were administered at baseline and immediately following the intervention period by trained assessors utilizing standardized procedures. General demographic, health-related data, and physical activity level, were collected through a structured questionnaire. Physical activity was assessed using the Physical Activity Scale for the Elderly (PASE), a validated questionnaire that evaluates physical activity in older adults over the preceding week, including occupational, household, and leisure-time activities, with higher scores indicating greater levels of physical activity. Cognitive functions were evaluated using a comprehensive test battery. Overall cognitive function was assessed using the the MoCA. The threshold score for cognitive impairment is set at 26 points, with an additional point awarded to individuals with an educational duration of 6 years or less. Memory function was assessed using the Huashan Version of the Auditory Verbal Learning Test (AVLT-H), which involves the presentation of 12 words (e.g., coat, trousers, headscarf) followed by three immediate recall trials, short-delay (5 min) and long-delay (20 min) recall trials, and concludes with a recognition test. Attention was measured using the Digit Span Test (DST), which includes forward and backward components and is scored based on the longest sequence of digits correctly repeated. Executive function was evaluated using the Trail Making Test B (TMT-B), which required participants to alternately connect numbers and symbols, with the time taken for completion recorded in seconds. Language function was assessed through the Boston Naming Test (BNT), in which participants were tasked with naming 30 object pictures, with a maximum possible score of 30 points. Visuospatial function was measured using the Clock Drawing Test (CDT), which employed a 4-point scoring system evaluating the drawing of a closed disk, correct number placement, completion of all 12 numbers, and accurate pointer positioning, with participants instructed to depict a clock face showing the time “1:50.” The sequence of cognitive tests was standardized across all participants and assessment sessions. Short rest intervals were incorporated between tests to mitigate the effects of fatigue.

### 2.5 Bias control and quality assurance

Cognitive assessments were conducted following standardized protocols to maintain consistency and comparability across participants and assessment time points. The randomization sequence was concealed from research staff, and outcome assessors were blinded to group assignments to minimize bias. Intervention staff underwent standardized training and adhered to uniform intervention manuals throughout the study. Data entry and management procedures were regularly monitored to ensure accuracy, and reasons for participant dropout were systematically documented.

### 2.6 Statistical analysis

Statistical analyses were performed utilizing SPSS software, version 25.0. The Shapiro–Wilk test was employed to assess the normality of continuous variables. Quantitative data conforming to a normal distribution were reported as mean ± standard deviation, whereas categorical data were presented as frequencies. For baseline group comparisons, continuous variables that satisfied normality assumptions were analyzed using one-way analysis of variance (ANOVA), and categorical variables were assessed via the chi-square test. Within-group changes from baseline to post-intervention were examined using paired t-tests. To assess the main effects of Baduanjin (yes/no) and cognitive training (yes/no), and their interaction, a 2 × 2 factorial ANOVA was conducted, with change scores (post-intervention minus baseline) as the dependent variable. Simple effects analysis with the Šidák correction was performed if a significant interaction effect was detected. All statistical tests were two-tailed, with the significance threshold set at *p* < 0.05.

## 3 Results

### 3.1 Participant characteristics at baseline

A total of 162 community-dwelling elderly individuals with normal cognitive function were randomly assigned to four groups: control (CON, *n* = 43), Baduanjin group (BG, *n* = 40), cognitive training (CT, *n* = 41), and combined intervention (BG + CT, *n* = 38). After 12 weeks, 138 participants completed the study (CON, *n* = 35; BG, *n* = 36; CT, *n* = 34; BG + CT, *n* = 33), with an overall attrition rate of 14.8%, primarily due to voluntary withdrawal, loss to follow-up, or health issues (Figure 1). Baseline demographic, cognitive, physical activity, and emotional characteristics were comparable across groups (*p* > 0.05; Table 1; Supplementary Table 1), confirming successful randomization.

**Table 1:** Participant characteristics at baseline.

Characteristics	CON (*n* = 35)	BG (*n* = 36)	CT (*n* = 34)	BG + CT (*n* = 33)	*F/χ2*	*p*
Age, years	66.2 ± 4.39	64.33 ± 4.09	66.06 ± 3.45	66.06 ± 4.63	*F* = 1.621	0.187
Gender (Male/Female)	18/17	17/19	16/18	16/17	*χ*^2^ = 0.172	0.982
Education, *n* (%)					*χ*^2^ = 5.766	0.450
<9 years	12 (34.3)	4 (11.1)	8 (23.5)	8 (24.2)		
9–12 years	15 (42.9)	22 (61.1)	16 (47.1)	16 (48.5)		
>12 years	8 (22.9)	10 (27.8)	10 (29.4)	9 (27.3)		
Physical activity level (PASE)	161.78 ± 28.48	158.29 ± 20.32	153.08 ± 22.50	155.18 ± 29.90	*F* = 0.762	0.517
Overall cognitive function (MoCA)	26.54 ± 0.81	26.56 ± 0.69	26.59 ± 0.70	26.52 ± 0.57	*F* = 0.063	0.979
Memory function						
Immediate memory	5.46 ± 0.78	5.27 ± 0.75	5.39 ± 0.94	5.42 ± 0.74	*F* = 0.372	0.773
Short-delay memory	5.17 ± 1.15	5.03 ± 0.84	5.09 ± 0.93	5.27 ± 0.63	*F* = 0.466	0.707
Long-delay memory	5.00 ± 1.06	4.89 ± 1.01	4.94 ± 0.81	4.91 ± 0.52	*F* = 0.106	0.956
Recognition function	10.17 ± 0.98	10.44 ± 1.18	10.24 ± 1.05	10.36 ± 1.17	*F* = 0.443	0.723
Attention						
Forward digit span	11.40 ± 1.40	11.64 ± 0.76	11.32 ± 1.25	11.52 ± 0.97	*F* = 0.529	0.663
Reverse digit span	6.80 ± 1.39	7.14 ± 1.29	6.68 ± 0.94	6.97 ± 1.42	*F* = 0.872	0.457
Executive function (TMT-B)^a^	168.34 ± 25.09	166.11 ± 17.58	163.82 ± 18.77	170.48 ± 29.20	*F* = 0.522	0.668
Language function (BNT)	25.37 ± 1.82	25.94 ± 1.55	25.44 ± 1.50	25.27 ± 1.21	*F* = 1.336	0.265
Visuospatial function (CDT)	2.97 ± 0.62	2.89 ± 0.62	3.05 ± 0.78	2.82 ± 0.67	*F* = 0.797	0.497

Continuous variables are presented as mean ± standard deviation and were compared using one-way ANOVA (reported as F); categorical variables are presented as count (*n*) with percentage in parentheses (%) and were compared using the chi-square test (reported as χ^2^). CON, control group; BG, Baduanjin group; CT, cognitive training group; BG+CT, combined group; PASE, the Physical Activity Scale for the Elderly; MoCA, Montreal Cognitive Assessment; TMT-B, Trail Making Test Part B; BNT, Boston Naming Test; CDT, Clock Drawing Test.

a TMT-B (Trail Making Test Part B) is reported in seconds; all other cognitive outcomes are reported in raw test scores.

### 3.2 Changes in global and domain-specific cognitive function following the intervention

Following the 12-week intervention, within-group analyses (Table 2; Supplementary Table 2) revealed distinct patterns of cognitive change. The CON group showed no significant improvements in global cognition (MoCA: 26.54 ± 0.81 *vs.* 26.63 ± 0.81, *p* = 0.373), whereas BG, CT, and BG + CT groups demonstrated significant increases in MoCA scores (*p* < 0.001), with the largest improvement observed in BG + CT. Memory analyses indicated that immediate and short-delay memory improved significantly in BG, CT, and BG + CT (*p* < 0.05), but long-delay memory improved only in BG + CT (*p* < 0.001). Recognition memory increased significantly in CON (*p* = 0.033) and in all intervention groups (*p* < 0.05). All intervention groups also showed improvements in attention, executive function, and visuospatial ability, as reflected by forward and reverse digit span, TMT-B completion time, and CDT scores, with the most pronounced gains in BG + CT (*p* < 0.01). Language function (BNT) improved significantly in CT and BG + CT (*p* < 0.001), while BG exhibited a marginal increase (*p* = 0.058). Regarding emotional outcomes, depression and anxiety scores decreased significantly in BG and BG + CT (*p* < 0.001), with no significant changes in CON or CT (*p* > 0.05, Supplementary Table 3).

**Table 2:** Cognitive function outcomes in each group before and after intervention.

Outcomes		CON (*n* = 35)	BG (*n* = 36)	CT (*n* = 34)	BG + CT (*n* = 33)
Overall cognitive function (MoCA)	Pre	26.54 ± 0.81	26.56 ± 0.69	26.59 ± 0.70	26.52 ± 0.57
	Post	26.63 ± 0.81	27.39 ± 0.60	27.35 ± 0.60	28.42 ± 0.61
	*t*	−0.902	−9.860	−8.990	−21.000
	*p*	0.373	<0.001	<0.001	<0.001
Memory function					
Immediate memory	Pre	5.46 ± 0.78	5.27 ± 0.75	5.39 ± 0.94	5.42 ± 0.74
	Post	5.62 ± 0.90	6.42 ± 1.02	6.35 ± 1.15	6.86 ± 0.83
	*t*	−1.495	−9.350	−7.599	−10.436
	*p*	0.144	<0.001	<0.001	<0.001
Short-delay memory	Pre	5.17 ± 1.15	5.03 ± 0.84	5.09 ± 0.93	5.27 ± 0.63
	Post	5.09 ± 1.12	5.58 ± 0.81	5.53 ± 0.93	5.91 ± 0.68
	*t*	0.828	−5.493	−3.651	−5.600
	*p*	0.413	<0.001	<0.001	<0.001
Long-delay memory	Pre	5.00 ± 1.06	4.89 ± 1.01	4.94 ± 0.81	4.91 ± 0.52
	Post	5.03 ± 1.22	5.08 ± 0.87	5.03 ± 1.06	5.70 ± 0.85
	*t*	−0.239	−1.420	−0.828	−5.520
	*p*	0.812	0.165	0.414	<0.001
Recognition	Pre	10.17 ± 0.98	10.44 ± 1.18	10.24 ± 1.05	10.36 ± 1.17
	Post	10.37 ± 0.91	10.69 ± 0.92	10.53 ± 1.02	10.82 ± 0.98
	*t*	−2.227	−2.311	−3.708	−4.629
	*p*	0.033	0.027	0.001	<0.001
Attention function					
Forward digit span	Pre	11.40 ± 1.40	11.64 ± 0.76	11.32 ± 1.25	11.52 ± 0.97
	Post	11.63 ± 1.35	12.14 ± 0.54	12.26 ± 0.83	12.63 ± 0.78
	*t*	−1.961	−3.550	−5.263	−9.250
	*p*	0.058	0.001	<0.001	<0.001
Reverse digit span	Pre	6.80 ± 1.39	7.14 ± 1.29	6.68 ± 0.94	6.97 ± 1.42
	Post	6.77 ± 1.42	8.17 ± 1.28	7.94 ± 1.25	8.45 ± 1.03
	*t*	0.442	−5.700	−7.270	−9.800
	*p*	0.661	<0.001	<0.001	<0.001
Executive function (TMT-B)^a^	Pre	168.34 ± 25.09	166.11 ± 17.58	163.82 ± 18.77	170.48 ± 29.20
	Post	165.74 ± 25.71	134.56 ± 17.56	143.82 ± 17.93	130.21 ± 24.66
	*t*	1.491	12.538	12.297	17.127
	*p*	0.145	<0.001	<0.001	<0.001
Language function (BNT)	Pre	25.37 ± 1.82	25.94 ± 1.55	25.44 ± 1.50	25.27 ± 1.21
	Post	25.46 ± 1.67	26.08 ± 1.40	26.32 ± 1.30	26.52 ± 1.09
	*t*	−1.358	−1.963	−10.771	−16.4
	*p*	0.183	0.058	<0.001	<0.001
Visuospatial function (CDT)	Pre	2.97 ± 0.62	2.89 ± 0.62	3.05 ± 0.78	2.82 ± 0.67
	Post	3.03 ± 0.38	3.31 ± 0.47	3.29 ± 0.68	3.79 ± 0.48
	*t*	−0.702	−5.000	−3.187	−8.750
	*p*	0.487	<0.001	0.003	<0.001

Data are presented as mean ± standard deviation. “Pre” and “Post” denote assessment before and after the intervention, respectively. “t” refers to the statistic from paired-sample *t*-tests. CON, control group; BG, Baduanjin group; CT, cognitive training group; BG+CT, combined group; MoCA, Montreal Cognitive Assessment; TMT-B, Trail Making Test Part B; BNT, Boston Naming Test; CDT, Clock Drawing Test.

a TMT-B (Trail Making Test Part B) is reported in seconds; all other cognitive outcomes are reported in raw test scores.

### 3.3 Interactive effects of Baduanjin exercise and cognitive training on cognitive outcomes

A 2 × 2 factorial ANOVA examining the main effects of Baduanjin and cognitive training revealed significant main effects of both interventions on global cognition (MoCA), memory (immediate, short-delay, long-delay), reverse digit span, executive function (TMT-B), language (BNT), and visuospatial function (CDT) (*p* < 0.05, partial *η^2^* range 0.047–0.558; Table 3). Interaction effects (BG × CT) were significant for MoCA (*η^2^* = 0.036, *p* = 0.027), memory subdomains (*η^2^* = 0.030–0.032, *p* < 0.05), reverse digit span (*η^2^* = 0.054, *p* = 0.006), TMT-B (*η^2^* = 0.031, *p* = 0.041), BNT (*η^2^* = 0.032, *p* = 0.038), and CDT (*η^2^* = 0.033, *p* = 0.035). For recognition memory, no significant main or interaction effects were observed. For forward digit span, a significant main effect of cognitive training was observed (*p* < 0.001; Table 3), while no significant main effect of Baduanjin or interaction was detected. For emotional outcomes (Supplementary Table 4), BG showed large main effects on depression (*η^2^* = 0.191, *p* < 0.01) and anxiety (*η^2^* = 0.320, *p* < 0.01), whereas CT had no significant effects (*p* > 0.05).

**Table 3:** Main and interactive effects of the interventions on changes in cognitive outcomes.

Outcomes	Effect	*F*	*p*	*η* ^2^	Effect Size Interpretation
Overall Cognitive Function (MoCA)	BG	112.958	<0.001	0.457	Very Large
	CT	97.163	<0.001	0.420	Very Large
	BG × CT	4.968	0.027	0.036	Small
Memory function					
Immediate memory	BG	34.650	<0.001	0.205	Large
	CT	19.151	<0.001	0.125	Medium/Large
	BG × CT	4.368	0.039	0.032	Small
Short-delay memory	BG	14.515	<0.001	0.098	Medium/Large
	CT	7.662	0.006	0.054	Medium
	BG × CT	4.128	0.044	0.030	Small
Long-delay memory	BG	11.553	0.001	0.079	Medium
	CT	6.578	0.011	0.047	Medium
	BG × CT	4.394	0.038	0.032	Small
Recognition	BG	1.229	0.270	0.009	–
	CT	2.476	0.118	0.018	–
	BG × CT	0.339	0.562	0.003	–
Attention function					
Forward digit span	BG	2.542	0.113	0.019	–
	CT	22.189	<0.001	0.142	Large
	BG × CT	0.104	0.747	0.001	–
Reverse digit span	BG	17.950	<0.001	0.118	Medium/Large
	CT	33.750	<0.001	0.201	Large
	BG × CT	7.703	0.006	0.054	Medium
Executive function (TMT-B)^a^	BG	137.128	<0.001	0.506	Very Large
	CT	38.596	<0.001	0.224	Large
	BG × CT	4.266	0.041	0.031	Small
Language function (BNT)	BG	8.002	0.005	0.056	Medium
	CT	169.192	<0.001	0.558	Very Large
	BG × CT	4.413	0.038	0.032	Small
Visuospatial function (CDT)	BG	38.675	<0.001	0.224	Large
	CT	17.279	<0.001	0.114	Medium/Large
	BG × CT	4.542	0.035	0.033	Small

This table presents the results of a 2 × 2 factorial analysis of variance (ANOVA). “Effect” indicates the factor tested: BG (main effect of Baduanjin), CT (main effect of cognitive training), and BG×CT (interaction effect). F and *p* values are reported for each effect. η^2^ represents partial eta squared and is reported as the measure of effect size (small effect: ≥0.01, medium effect: ≥0.06, large effect: ≥0.14). Dashes (−) indicate effect sizes below the threshold for a “Small” interpretation (η^2^ < 0.01). BG, Baduanjin; CT, cognitive training; MoCA, Montreal Cognitive Assessment; TMT-B, Trail Making Test Part B; BNT, Boston Naming Test; CDT, Clock Drawing Test.

a TMT-B (Trail Making Test Part B) is reported in seconds; all other cognitive outcomes are reported in raw test scores.

### 3.4 Simple effects analyses of significant intervention interactions

Simple effects analyses confirmed that the combined BG + CT intervention outperformed the single interventions across multiple domains (Table 4). MoCA improvement in BG + CT (1.91 ± 0.09) was significantly greater than in BG (0.83 ± 0.09) and CT (0.77 ± 0.09) (*p* < 0.001). For long-delay memory, neither Baduanjin nor cognitive training alone produced significant gains relative to CON, whereas BG + CT significantly outperformed both single interventions (*p* < 0.01). Executive function (TMT-B), language (BNT), and visuospatial ability (CDT) also improved more in BG + CT than in either single intervention (*p* < 0.01). Reverse digit span improvement in BG + CT was significantly greater than BG (*p* = 0.034) but not CT (*p* = 0.310). These findings indicate that combining Baduanjin with cognitive training produced the largest gains across most cognitive domains.

**Table 4:** Simple effects of the interventions on cognitive outcomes.

Outcomes	CON	*VS.*	CT	BG	*VS.*	BG + CT	CON	*VS.*	BG	CT	*VS.*	BG + CT
MoCA	0.086 ± 0.088		0.765 ± 0.090	0.833 ± 0.087		1.909 ± 0.091	0.086 ± 0.088		0.833 ± 0.087	0.765 ± 0.090		1.909 ± 0.091
*Δ*		0.679			1.076			0.748			1.144	
*F*		29.120			72.973			36.328			80.321	
*p*		<0.001			<0.001			<0.001			<0.001	
Immediate memory	0.161 ± 0.123		0.961 ± 0.125	1.148 ± 0.121		1.431 ± 0.126	0.161 ± 0.123		1.148 ± 0.121	0.961 ± 0.125		1.431 ± 0.126
*Δ*		0.800			0.283			0.987			0.470	
*F*		20.922			2.611			32.761			7.004	
*p*		<0.001			0.108			<0.001			<0.001	
Short-Delay memory	−0.086 ± 0.109		0.441 ± 0.111	0.556 ± 0.107		0.636 ± 0.112	−0.086 ± 0.109		0.556 ± 0.107	0.441 ± 0.111		0.636 ± 0.112
*Δ*		0.527			0.081			0.641			0.195	
*F*		11.529			0.271			17.572			1.536	
*p*		0.001			0.604			<0.001			0.217	
Long-Delay memory	0.029 ± 0.126		0.088 ± 0.128	0.194 ± 0.125		0.788 ± 0.130	0.029 ± 0.126		0.194 ± 0.125	0.088 ± 0.128		0.788 ± 0.130
*Δ*		0.059			0.593			0.166			0.700	
*F*		0.110			10.853			0.874			14.673	
*p*		0.741			0.001			0.352			<0.001	
Reverse digit span	−0.029 ± 0.149		1.265 ± 0.152	1.028 ± 0.147		1.485 ± 0.154	−0.029 ± 0.149		1.028 ± 0.147	1.265 ± 0.152		1.485 ± 0.154
*Δ*		1.293			0.457			1.056			0.220	
*F*		36.882			4.599			25.320			1.038	
*p*		<0.001			0.034			<0.001			0.310	
TMT-B^a^	−2.600 ± 2.086		−20.000 ± 2.116	−31.556 ± 2.057		−40.273 ± 2.148	−2.600 ± 2.086		−31.556 ± 2.057	−20.000 ± 2.116		−40.273 ± 2.148
*Δ*		−17.400			−8.717			−8.717			−20.270	
*F*		34.292			8.592			97.717			45.200	
*p*		<0.001			0.004			<0.001			<0.001	
BNT	0.086 ± 0.072		0.882 ± 0.074	0.139 ± 0.071		1.242 ± 0.075	0.086 ± 0.072		0.139 ± 0.071	0.882 ± 0.074		1.242 ± 0.075
*Δ*		0.797			1.104			0.053			0.360	
*F*		59.527			114.033			0.273			11.808	
*p*		<0.001			<0.001			0.602			<0.001	
CDT	0.057 ± 0.087		0.235 ± 0.089	0.417 ± 0.086		0.970 ± 0.090	0.057 ± 0.087		0.417 ± 0.086	0.235 ± 0.089		0.970 ± 0.090
*Δ*		0.178			0.553			0.360			0.734	
*F*		2.053			19.752			8.604			33.880	
*p*		0.154			<0.001			0.004			<0.001	

Data are presented as mean change score ± standard error, the mean difference between groups (Δ), and the results of one-way ANOVA (F and P) for each pairwise comparison. “Δ” represents the difference in change scores between two groups. The *p* values reported are Šidák-corrected for multiple comparisons. CON, control group; BG, Baduanjin group; CT, cognitive training group; BG+CT, combined group; MoCA, Montreal Cognitive Assessment; TMT-B, Trail Making Test Part B; BNT, Boston Naming Test; CDT, Clock Drawing Test.

a TMT-B (Trail Making Test Part B) is reported in seconds; all other cognitive outcomes are reported in raw test scores.

## 4 Discussion

This study investigated the effects of a 12-week Baduanjin exercise, cognitive training, and their combined application on global cognitive function and domain-specific cognitive performance in cognitively normal older adults. The core finding of this research is that while both Baduanjin and cognitive training independently resulted in significant improvements across multiple cognitive domains, the combined intervention was associated with broader cognitive benefits, particularly in global cognitive function, long-delay memory, executive function, language function, and visuospatial function. This study advances current understanding of non-pharmacological strategies for preventing cognitive decline by focusing on cognitively intact older adults, a demographic that is underrepresented in existing intervention research.

The superior efficacy of the combined Baduanjin and cognitive training intervention likely stems from the complementary neurobiological pathways through which Baduanjin and cognitive training collectively promote brain health. Baduanjin, as a traditional form of physical and mental exercise, harmoniously combines mental focus, body posture, movement, and breath control to enhance cognitive functions such as concentration, memory, executive function, and visuospatial skills (Koh, 1982). This coordinated practice lays a solid physiological foundation for cognitive improvement. The mechanisms involve enhancing cerebral blood flow (Lin et al., 2023), boosting BDNF levels (Zhou et al., 2025), modulating structural plasticity in hippocampal subregions (Wan et al., 2022), and improving resting-state functional connectivity between the hippocampus and medial prefrontal cortex (Tao et al., 2016). Additionally, Baduanjin influences intrinsic functional connectivity and affects the norepinephrine and dopamine systems (Liu et al., 2021). In contrast, cognitive training leverages the brain’s neuroplasticity to stimulate targeted remodeling of specific brain regions and networks (Wu et al., 2023), manifested as increased gray matter (Engvig et al., 2014), enhanced white matter fiber integrity (Dai et al., 2024), and strengthened functional connectivity in cognition-related brain networks (Anderson-Hanley et al., 2018; Bigliassi et al., 2025; Faraza et al., 2020; Hardcastle et al., 2022). The observed combined effects of Baduanjin and cognitive training may stem from the former establishing an improved foundation for overall brain health, enabling the latter to perform targeted fine-tuning of neural circuits. The effectiveness of the combined intervention of Baduanjin and cognitive training has been validated in elderly diabetic patients with cognitive frailty (Liu et al., 2023).

It is important to note that the small but statistically significant interaction effect of the combined Baduanjin and cognitive training on global cognition (MoCA) is both expected and meaningful in the context of a preventive intervention for cognitively normal older adults. This outcome is likely attributable to their already high baseline cognitive function, coupled with the relatively short 12-week intervention period, which inherently limits the scope for substantial cognitive gains. Therefore, detecting any positive synergistic effect under these conditions underscores the potential efficacy and sensitivity of the combined approach.

Both BG and CT effectively improved immediate memory, short-delay memory, and recognition. However, only BG + CT resulted in a significant improvement in long-delay memory, which stands out as one of the study’s noteworthy findings. This benefit likely arises from the synergy between Baduanjin enhancing brain neurophysiological function and cognitive training reshaping neural networks for long-term memory (Zotcheva et al., 2022). Their combination creates a “neurophysiological optimization + task-specific training” model that uniquely supports memory consolidation (Marin Vargas et al., 2024). In the context of immediate and short-delay memory, the lack of additional benefit from BG + CT in these domains could suggest a ceiling effect at the behavioral level. Recognition memory improved across all groups, likely due to test familiarity from repeated assessments. This familiarity may have caused a ceiling effect, especially with high baseline scores, limiting the clinical interpretation of the intervention’s impact. Future studies should adopt more challenging tasks to better capture intervention effects on memory retrieval (Ferguson, 2021).

All intervention groups showed significant improvements in reverse digit span (attention) and TMT-B performance (executive function), with BG + CT outperforming single interventions. The practice of Baduanjin necessitates mental tranquility and focused intention, thereby augmenting attentional concentration. During practice, practitioners must standardize their movements and consciously suppress unnecessary or incorrect actions, relying on the inhibition function, a key part of executive function (Bigliassi et al., 2025; Wang et al., 2021; Wang et al., 2024). Combined with cognitive training demanding high-level cognitive control (e.g., reasoning, puzzle-solving), this forms a “dual-task” paradigm that strengthens prefrontal cortex-dominated cognitive control abilities (Duverne and Koechlin, 2017; Luciana and Collins, 2022; Miller, 2000). For forward digit span, no significant interaction effect was observed, suggesting basic attentional processes can be sufficiently improved by single interventions. In terms of language function, CT and BG + CT significantly improved BNT scores, while BG alone showed only a marginal effect, indicating language retrieval relies heavily on specialized cortical networks activated by targeted cognitive training (Jones et al., 2021). Nevertheless, BG + CT achieved the largest BNT gains, suggesting Baduanjin indirectly supports language performance by enhancing cognitive vitality and reducing anxiety. For visuospatial function, BG and BG + CT produced significant CDT score improvements, while CT alone had limited effects. Baduanjin’s directional shift movements, such as those performed in “Drawing a Bow to Both Sides Like Shooting an Eagle” (which involves coordinated torso rotation and contralateral limb movement), stimulate spatial perception and visuomotor integration, complementing cognitive training’s focus on visual planning (Lin L. Y. et al., 2022). CT’s limited effects may relate to insufficient task specificity or insufficient duration of the intervention (Orgeta et al., 2020). A secondary finding is that BG alone or combined with CT significantly reduced depression and anxiety scores. The reduction in negative affect may have contributed to cognitive gains by freeing up cognitive resources otherwise occupied by emotional regulation, thereby creating a more conducive state for cognitive training to take effect (Long et al., 2020).

It is necessary to acknowledge several limitations. First, participants were recruited from only one region, which may limit the generalizability of the funding to different populations. Second, the study lacked long-term follow-up, making it impossible to verify the sustainability of intervention effects, which is critical for a preventive strategy targeting cognitive decline. Third, participants in the combined intervention group received a higher overall intervention dose than those in the single-intervention groups. Therefore, the observed superiority of the combined intervention should be interpreted with caution due to potential differences in intervention exposure. Fourth, participants’ unsupervised physical activity outside the intervention sessions was not objectively monitored, which may have introduced confounding effects. Finally, neuroimaging and biomarker data were not collected, limiting the ability to directly validate the proposed neural mechanisms.

Future research should prolong intervention and follow-up periods to explore whether BG + CT delays the transition from normal cognition to MCI. Multi-modal assessments should be incorporated to elucidate the neural underpinnings of combined intervention benefits. Large-scale multi-center trials with diverse cohorts are needed to strengthen generalizability, while accelerometers or activity logs should be used to objectively capture all physical activity throughout the intervention period.

In conclusion, the 12-week Baduanjin, cognitive training, and combined interventions all improved global cognitive function and multiple specific cognitive domains in cognitively normal older adults. Critically, the combined intervention yielded greater improvements in global cognition, long-delay memory, executive function, language function, and visuospatial abilities than the single interventions. These findings underscore the importance of multidimensional strategies that integrate physical exercise with cognitive stimulation for preventing age-related cognitive decline. Given its feasibility, safety, and cost-effectiveness as a non-pharmacological intervention, the combination of Baduanjin and cognitive training should be prioritized in community-based geriatric health promotion programs. This approach holds promise for addressing the growing challenge of cognitive decline in aging populations and for improving brain health and quality of life among older adults.

## Data Availability

The original contributions presented in the study are included in the article/Supplementary material, further inquiries can be directed to the corresponding author.

## References

[ref1] Anderson-Hanley C. Barcelos N. M. Zimmerman E. A. Gillen R. W. Dunnam M. Cohen B. D. . (2018). The aerobic and cognitive exercise study (ACES) for community-dwelling older adults with or at-risk for mild cognitive impairment (MCI): neuropsychological, neurobiological and neuroimaging outcomes of a randomized clinical trial. Front. Aging Neurosci. 10:76. doi: 10.3389/fnagi.2018.00076, 29780318 PMC5945889

[ref2] Bigliassi M. Cabral D. F. Evans A. C. (2025). Improving brain health via the central executive network. J. Physiol. 604, 2375–2399. doi: 10.1113/jp287099, 39856810

[ref3] Castellote-Caballero Y. Carcelén Fraile M. D. C. Aibar-Almazán A. Afanador-Restrepo D. F. González-Martín A. M. (2024). Effect of combined physical-cognitive training on the functional and cognitive capacity of older people with mild cognitive impairment: a randomized controlled trial. BMC Med. 22:281. doi: 10.1186/s12916-024-03469-x, 38972988 PMC11229192

[ref4] Chen Y. L. Tseng C. H. Lin H. T. Wu P. Y. Chao H. C. (2023). Dual-task multicomponent exercise-cognitive intervention improved cognitive function and functional fitness in older adults. Aging Clin. Exp. Res. 35, 1855–1863. doi: 10.1007/s40520-023-02481-0, 37418224

[ref5] Cheng S. T. (2016). Cognitive reserve and the prevention of dementia: the role of physical and cognitive activities. Curr. Psychiatry Rep. 18:85. doi: 10.1007/s11920-016-0721-2, 27481112 PMC4969323

[ref6] Dai X. Liu S. Li Y. Long S. Li X. Chen C. . (2024). White matter plasticity underpins cognitive gains after multidomain adaptive computerized cognitive training. J. Gerontol. A Biol. Sci. Med. Sci. 79:glae046. doi: 10.1093/gerona/glae046, 38387014

[ref7] Dar N. J. Currais A. Taguchi T. Andrews N. Maher P. (2025). Cannabinol (CBN) alleviates age-related cognitive decline by improving synaptic and mitochondrial health. Redox Biol. 84:103692. doi: 10.1016/j.redox.2025.103692, 40412024 PMC12150171

[ref8] Duverne S. Koechlin E. (2017). Rewards and cognitive control in the human prefrontal cortex. Cereb. Cortex 27, 5024–5039. doi: 10.1093/cercor/bhx210, 28922835

[ref9] Engvig A. Fjell A. M. Westlye L. T. Skaane N. V. Dale A. M. Holland D. . (2014). Effects of cognitive training on gray matter volumes in memory clinic patients with subjective memory impairment. J. Alzheimer's Dis 41, 779–791. doi: 10.3233/jad-131889, 24685630

[ref10] Fang P. Gao Y. Li Y. Li C. Zhang T. Wu L. . (2024). Effects of computerized working memory training on neuroplasticity in healthy individuals: a combined neuroimaging and neurotransmitter study. NeuroImage 298:120785. doi: 10.1016/j.neuroimage.2024.120785, 39154869

[ref11] Faraza S. Waldenmaier J. Dyrba M. Wolf D. Fellgiebel A. Teipel S. J. (2020). Dorsolateral prefrontal functional connectivity predicts transfer of working memory gains after cognitive training: prevention (nonpharmacological) / cognitive interventions. Alzheimers Dement. 16:e042981. doi: 10.1002/alz.042981

[ref12] Ferguson C. (2021). A network psychometric approach to neurocognition in early Alzheimer's disease. Cortex 137, 61–73. doi: 10.1016/j.cortex.2021.01.002, 33607345

[ref13] Hardcastle C. Hausman H. K. Kraft J. N. Albizu A. O’Shea A. Boutzoukas E. M. . (2022). Proximal improvement and higher-order resting state network change after multidomain cognitive training intervention in healthy older adults. GeroScience 44, 1011–1027. doi: 10.1007/s11357-022-00535-1, 35258771 PMC9135928

[ref14] Herrmann M. J. Wuttke A. Breuninger L. Eff J. Ettlinger S. Fischer M. . (2025). Functional near-infrared spectroscopy and vagus somatosensory evoked potentials add to the power of established parameters such as poor cognitive performance, dsyosmia and APOe genotype to predict cognitive decline over 8 years in the elderly. J. Neural Transm. (Vienna) 132, 455–468. doi: 10.1007/s00702-024-02859-y, 39535568 PMC11870936

[ref15] Hong Y. Tian Z. Ji Z. Yang J. Wang C. (2024). A systematic review of the effect and mechanism of Daoyin therapy on improving mild cognitive impairment in older adults. Ageing Res. Rev. 101:102526. doi: 10.1016/j.arr.2024.102526, 39368667

[ref16] Jakovljevic M. M. Netz Y. Buttigieg S. C. Adany R. Laaser U. Varjacic M. (2018). Population aging and migration - history and UN forecasts in the EU-28 and its east and south near neighborhood - one century perspective 1950-2050. Glob. Health 14:30. doi: 10.1186/s12992-018-0348-7, 29548339 PMC5857107

[ref17] Jones K. O. Lopes S. S. Kelly C. Welsh R. S. Chen L. Wilson M. . (2021). A qualitative study on participants' experiences with a community-based mindful walking intervention and mobile device activity measurement. Complement. Ther. Med. 57:102640. doi: 10.1016/j.ctim.2020.102640, 33388390

[ref18] Kang H. (2021). Sample size determination and power analysis using the G*power software. J Educ Eval Health Prof 18:17. doi: 10.3352/jeehp.2021.18.17, 34325496 PMC8441096

[ref19] Kauppi K. Rönnlund M. Nordin Adolfsson A. Pudas S. Adolfsson R. (2020). Effects of polygenic risk for Alzheimer's disease on rate of cognitive decline in normal aging. Transl. Psychiatry 10:250. doi: 10.1038/s41398-020-00934-y, 32709845 PMC7381667

[ref20] Koh T. C. (1982). Baduanjin -- an ancient Chinese exercise. Am. J. Chin. Med. 10, 14–21. doi: 10.1142/s0192415x8200004x 7183203

[ref21] Lin L. Y. Chi I. J. Sung Y. S. (2022). Mediating effect of sequential memory on the relationship between visual-motor integration and self-care performance in young children with autism spectrum disorder. Front. Psychol. 13:988493. doi: 10.3389/fpsyg.2022.988493, 36275205 PMC9583898

[ref22] Lin S. Guo J. Nie P. Chen X. Guo J. Lin N. . (2022). Baduanjin for executive function in patients with mild cognitive impairment: a systematic review and meta-analysis. Complement. Ther. Clin. Pract. 49:101626. doi: 10.1016/j.ctcp.2022.101626, 35785625

[ref23] Lin H. Ye Y. Wan M. Qiu P. Xia R. Zheng G. (2023). Effect of Baduanjin exercise on cerebral blood flow and cognitive frailty in the community older adults with cognitive frailty: a randomized controlled trial. J. Exerc. Sci. Fit. 21, 131–137. doi: 10.1016/j.jesf.2022.12.001, 36606263 PMC9791406

[ref24] Lisko I. Kulmala J. Annetorp M. Ngandu T. Mangialasche F. Kivipelto M. (2021). How can dementia and disability be prevented in older adults: where are we today and where are we going? J. Intern. Med. 289, 807–830. doi: 10.1111/joim.13227, 33314384 PMC8248434

[ref25] Liu J. Tao J. Xia R. Li M. Huang M. Li S. . (2021). Mind-body exercise modulates locus Coeruleus and ventral tegmental area functional connectivity in individuals with mild cognitive impairment. Front. Aging Neurosci. 13:646807. doi: 10.3389/fnagi.2021.646807, 34194314 PMC8236862

[ref26] Liu Y. Xiaoyun W. Huinan Y. A. N. (2023). Intervention of Baduanjin combined with cognitive training on cognitive frailty in elderly diabetic patients: a clinical study. Chin. Gen. Pract. 26, 2848–2853. doi: 10.12114/j.issn.1007-9572.2023.0148

[ref27] Liu-Ambrose T. Barha C. Falck R. S. (2019). Active body, healthy brain: exercise for healthy cognitive aging. Int. Rev. Neurobiol. 147, 95–120. doi: 10.1016/bs.irn.2019.07.004, 31607364

[ref28] Livingston G. Huntley J. Sommerlad A. Ames D. Ballard C. Banerjee S. . (2020). Dementia prevention, intervention, and care: 2020 report of the lancet commission. Lancet 396, 413–446. doi: 10.1016/s0140-6736(20)30367-6, 32738937 PMC7392084

[ref29] Long Q. Hu N. Li H. Zhang Y. Yuan J. Chen A. (2020). Suggestion of cognitive enhancement improves emotion regulation. Emotion 20, 866–873. doi: 10.1037/emo0000629, 31282698

[ref30] Luciana M. Collins P. F. (2022). Neuroplasticity, the prefrontal cortex, and psychopathology-related deviations in cognitive control. Annu. Rev. Clin. Psychol. 18, 443–469. doi: 10.1146/annurev-clinpsy-081219-111203, 35534121

[ref31] Marin Vargas A. Bisi A. Chiappa A. S. Versteeg C. Miller L. E. Mathis A. (2024). Task-driven neural network models predict neural dynamics of proprioception. Cell 187, 1745–1761.e19. doi: 10.1016/j.cell.2024.02.036, 38518772

[ref32] Miller E. K. (2000). The prefrontal cortex and cognitive control. Nat. Rev. Neurosci. 1, 59–65. doi: 10.1038/35036228, 11252769

[ref33] Orgeta V. McDonald K. R. Poliakoff E. Hindle J. V. Clare L. Leroi I. (2020). Cognitive training interventions for dementia and mild cognitive impairment in Parkinson’s disease. Cochrane Database Syst. Rev. 2020:Cd011961. doi: 10.1002/14651858.CD011961.pub2, 32101639 PMC7043362

[ref34] Pieramico V. Esposito R. Sensi F. Cilli F. Mantini D. Mattei P. A. . (2012). Combination training in aging individuals modifies functional connectivity and cognition, and is potentially affected by dopamine-related genes. PLoS One 7:e43901. doi: 10.1371/journal.pone.0043901, 22937122 PMC3429431

[ref35] Reijnders J. van Heugten C. van Boxtel M. (2013). Cognitive interventions in healthy older adults and people with mild cognitive impairment: a systematic review. Ageing Res. Rev. 12, 263–275. doi: 10.1016/j.arr.2012.07.003, 22841936

[ref36] Shen Y. Z. Chen F. Yu J. W. Zhang Y. Lu L. X. Huo Y. L. . (2024). Review of Baduanjin and resistance exercise for the mental health of patients with hematologic malignancies. World J Psychiatry 14, 1165–1173. doi: 10.5498/wjp.v14.i8.1165, 39165558 PMC11331390

[ref37] Sun M. K. Alkon D. L. (2024). Alzheimer's therapeutic development: shifting neurodegeneration to neuroregeneration. Trends Pharmacol. Sci. 45, 197–209. doi: 10.1016/j.tips.2024.01.012, 38360510 PMC10939773

[ref38] Sung C. M. Jen H. J. Liu D. Kustanti C. Y. Chu H. Chen R. . (2023). The effect of cognitive training on domains of attention in older adults with mild cognitive impairment and mild dementia: a meta-analysis of randomised controlled trials. J. Glob. Health 13:04078. doi: 10.7189/jogh.13.04078, 37387539 PMC10312045

[ref39] Tao J. Liu J. Egorova N. Chen X. Sun S. Xue X. . (2016). Increased Hippocampus-medial prefrontal cortex resting-state functional connectivity and memory function after tai Chi Chuan practice in elder adults. Front. Aging Neurosci. 8:25. doi: 10.3389/fnagi.2016.00025, 26909038 PMC4754402

[ref40] Turnbull A. Seitz A. Tadin D. Lin F. V. (2022). Unifying framework for cognitive training interventions in brain aging. Ageing Res. Rev. 81:101724. doi: 10.1016/j.arr.2022.101724, 36031055 PMC10681332

[ref41] van der Flier W. M. de Vugt M. E. Smets E. M. A. Blom M. Teunissen C. E. (2023). Towards a future where Alzheimer's disease pathology is stopped before the onset of dementia. Nat. Aging 3, 494–505. doi: 10.1038/s43587-023-00404-2, 37202515

[ref42] Wan M. Xia R. Lin H. Ye Y. Qiu P. Zheng G. (2022). Baduanjin exercise modulates the hippocampal subregion structure in community-dwelling older adults with cognitive frailty. Front. Aging Neurosci. 14:956273. doi: 10.3389/fnagi.2022.956273, 36600804 PMC9806122

[ref43] Wang X. Wu J. Ye M. Wang L. Zheng G. (2021). Effect of Baduanjin exercise on the cognitive function of middle-aged and older adults: a systematic review and meta-analysis. Complement. Ther. Med. 59:102727. doi: 10.1016/j.ctim.2021.102727, 33933577

[ref44] Wang X. Wu J. Zhang H. Zheng G. (2024). Effect of Baduanjin exercise on executive function in older adults with cognitive frailty: a randomized controlled trial. Clin. Rehabil. 38, 510–519. doi: 10.1177/02692155231215891, 38092741

[ref45] Wu Y. Xu L. Wu Z. Cao X. Xue G. Wang Y. . (2023). Computer-based multiple component cognitive training in children with ADHD: a pilot study. Child Adolesc. Psychiatry Ment. Health 17:9. doi: 10.1186/s13034-022-00553-z, 36647166 PMC9843988

[ref46] Xue D. Li P. W. C. Yu D. S. F. Lin R. S. Y. (2023). Combined exercise and cognitive interventions for adults with mild cognitive impairment and dementia: a systematic review and network meta-analysis. Int. J. Nurs. Stud. 147:104592. doi: 10.1016/j.ijnurstu.2023.104592, 37769394

[ref47] Ye M. Wang L. Xiong J. Zheng G. (2021). The effect of mind-body exercise on memory in older adults: a systematic review and meta-analysis. Aging Clin. Exp. Res. 33, 1163–1173. doi: 10.1007/s40520-020-01557-5, 32329024

[ref48] Yu L. Liu F. Nie P. Shen C. Chen J. Yao L. (2021). Systematic review and meta-analysis of randomized controlled trials assessing the impact of Baduanjin exercise on cognition and memory in patients with mild cognitive impairment. Clin. Rehabil. 35, 492–505. doi: 10.1177/0269215520969661, 33143442

[ref49] Zhang M. Jia J. Yang Y. Zhang L. Wang X. (2023). Effects of exercise interventions on cognitive functions in healthy populations: a systematic review and meta-analysis. Ageing Res. Rev. 92:102116. doi: 10.1016/j.arr.2023.102116, 37924980

[ref50] Zhang R. Liu S. Mousavi S. M. (2024). Cognitive dysfunction and exercise: from epigenetic to genetic molecular mechanisms. Mol. Neurobiol. 61, 6279–6299. doi: 10.1007/s12035-024-03970-7, 38286967

[ref51] Zhou Z. Wang J. Kong F. Zhang Q. (2025). Effect of Baduanjin exercise in a blended online-offline model on cognitive function and peripheral blood BDNF levels in older adults. Front. Physiol. 16:1573674. doi: 10.3389/fphys.2025.1573674, 40606229 PMC12215356

[ref52] Zotcheva E. Håberg A. K. Wisløff U. Salvesen Ø. Selbæk G. Stensvold D. . (2022). Effects of 5 years aerobic exercise on cognition in older adults: the generation 100 study: a randomized controlled trial. Sports Med. 52, 1689–1699. doi: 10.1007/s40279-021-01608-5, 34878637 PMC9213353

