# Supplementary material for: Effects of combined Baduanjin and cognitive training on cognitive function in cognitively normal older adults
Source: Front Psychol. 2026 Aug 14;17:1903040. doi: 10.3389/fpsyg.2026.1903040 (PMC13521854; doi:10.3389/fpsyg.2026.1903040)
Supplement: Supplementary file 1 [file Table_1.docx]

**Supplementary Table 1.** Baseline demographic and clinical characteristics of the participants by group.

| Characteristics | CON  （n=35） | BG  (n=36) | CT  （n=34） | BG+CT  （n=33） | *F* | *p* |
| --- | --- | --- | --- | --- | --- | --- |
| Depression | 2.69±1.28 | 2.00±1.43 | 2.50±1.29 | 2.61±1.09 | 2.054 | 0.109 |
| Anxiety | 32.71±4.72 | 33.82±4.99 | 34.08±5.63 | 33.41±5.70 | 0.450 | 0.718 |

*Note.* Continuous variables are presented as mean ± SD and were compared using one-way ANOVA; the *F* statistic is reported.

Abbreviations: CON, control group; BG, Baduanjin group; CT, cognitive training group; BG+CT, combined group.

**Supplementary Table 2.** *Changes in Cognitive and Clinical Outcomes Following the Intervention (Δ = Post - Pre)*

| Outcomes | CON(n=35) | BG (n=36) | CT(n=34) | BG+CT(n=33) |
| --- | --- | --- | --- | --- |
| Overall Cognitive Function (MoCA) | 0.086±0.562 | 0.833±0.507 | 0.765±0.496 | 1.909±0.522 |
| Memory Function |  |  |  |  |
| Immediate Memory | 0.161±0.639 | 1.148±0.737 | 0.962±0.738 | 1.431±0.788 |
| Short-Delay Memory | -0.086±0.612 | 0.556±0.607 | 0.441±0.705 | 0.636±0.653 |
| Long-Delay Memory | 0.029±0.707 | 0.194±0.822 | 0.088±0.621 | 0.788±0.820 |
| Recognition | 0.200±0.531 | 0.250±0.649 | 0.294±0.463 | 0.455±0.564 |
| Attention Function |  |  |  |  |
| Forward Sequence | 0.229±0.690 | 0.500±0.845 | 0.941±1.043 | 1.121±0.696 |
| Reverse Sequence | -0.029±0.382 | 1.028±1.082 | 1.265±1.024 | 1.485±0.870 |
| Executive Function (TMT-B)^a^ | -2.600±10.313 | -31.556±15.101 | -20.000±9.484 | -40.273±13.508 |
| Language Function (BNT) | 0.086±0.374 | 0.139±0.425 | 0.882±0.478 | 1.242±0.435 |
| Visuospatial Function (CDT) | 0.057±0.482 | 0.417±0.500 | 0.235±0.431 | 0.970±0.637 |
| Depression | -0.086±1.337 | -1.000±1.192 | -0.118±0.686 | -1.364±1.168 |
| Anxiety | 0.437±3.501 | -5.521±4.069 | 0.243±4.252 | -4.735±4.305 |

*Note.* Data are presented as mean change score ± standard error. *Δ*= post - pre. Abbreviations: CON, control group; BG, Baduanjin group; CT, cognitive training group; BG+CT, combined group; MoCA, Montreal Cognitive Assessment; TMT-B, Trail Making Test Part B; BNT, Boston Naming Test; CDT, Clock Drawing Test.

^a^: TMT-B (Trail Making Test Part B) is reported in seconds; all other cognitive outcomes are reported in raw test scores.

**Supplementary Table 3.** *Depression and Anxiety Outcomes in Each Group Before and After Intervention*

| Outcomes | | CON(n=35) | BG (n=36) | CT(n=34) | BG+CT(n=33) |
| --- | --- | --- | --- | --- | --- |
| Depression | Pre | 2.69±1.28 | 2.00±1.43 | 2.50±1.29 | 2.61±1.09 |
|  | Post | 2.60±1.01 | 1.00±0.86 | 2.38±1.04 | 1.24±0.87 |
|  | *t* | 0.379 | 5.020 | 1.000 | 6.708 |
|  | *p* | 0.707 | <0.001 | 0.325 | <0.001 |
| Anxiety | Pre | 32.71±4.72 | 33.82±4.99 | 34.08±5.63 | 33.41±5.70 |
|  | Post | 33.14±3.04 | 28.30±2.20 | 34.32±4.03 | 28.67±3.29 |
|  | *t* | -0.739 | 8.142 | -0.333 | 6.318 |
|  | *p* | 0.465 | <0.001 | 0.741 | <0.001 |

*Note.* Data are presented as mean ± standard deviation. “Pre” and “Post” denote assessment before and after the intervention, respectively. “*t*” refers to the statistic from paired-sample *t*-tests. Abbreviations: CON, control group; BG, Baduanjin group; CT, cognitive training group; BG+CT, combined group.

**Supplementary Table 4**

*Main and Interactive Effects of the Interventions on Depression and Anxiety Scores*

| Cognitive Outcome | Groups | *F* | *p* | Partial *η*² |
| --- | --- | --- | --- | --- |
| Depression | BG | 31.711 | <0.001 | 0.191 |
|  | CT | 1.063 | 0.304 | 0.008 |
|  | BG+CT | 0.748 | 0.389 | 0.006 |
| Anxiety | BG | 63.167 | <0.001 | 0.320 |
|  | CT | 0.185 | 0.668 | 0.001 |
|  | BG+CT | 0.508 | 0.477 | 0.004 |

*Note.*This table presents the results of a 2×2 factorial analysis of variance (ANOVA). “Effect Source” indicates the factor tested: BG (main effect of Baduanjin), CT (main effect of cognitive training), and BG×CT (interaction effect). *F* and *p* values are reported for each effect. *η*² represents partial eta squared and is reported as the measure of effect size (small effect: ≥ 0.01, medium effect: ≥ 0.06, large effect: ≥ 0.14). Abbreviations: CON, Control Group; BG, Baduanjin Group; CT, Cognitive Training Group; BG+CT, Combined Group.
